# Identification of Genes Putatively Involved in Chitin Metabolism and Insecticide Detoxification in the Rice Leaf Folder (*Cnaphalocrocis medinalis*) Larvae through Transcriptomic Analysis

**DOI:** 10.3390/ijms160921873

**Published:** 2015-09-10

**Authors:** Hai-Zhong Yu, De-Fu Wen, Wan-Lin Wang, Lei Geng, Yan Zhang, Jia-Ping Xu

**Affiliations:** 1 School of Life Sciences, Anhui Agricultural University, Hefei 230036, China; E-Mails: yuhaizhong1988@163.com (H.-Z.Y.); wendefu1@163.com (D.-F.W.); andrewfredrick@163.com (L.G.); 2 Rice Research Institute, Anhui Academy of Agricultural Sciences, Hefei 230031, China; E-Mail: wangwanlin0551@163.com; 3 Institute of Sericulture, Anhui Academy of Agricultural Sciences, Hefei 230061, China; E-Mail: zhangyanhao6667@163.com

**Keywords:** transcriptome, *Cnaphalocrocis medinalis*, chitin metabolism, insecticide detoxification, insecticide target

## Abstract

The rice leaf roller (*Cnaphalocrocis medinalis*) is one of the most destructive agricultural pests. Due to its migratory behavior, it is difficult to control worldwide. To date, little is known about major genes of *C. medinalis* involved in chitin metabolism and insecticide detoxification. In order to obtain a comprehensive genome dataset of *C. medinalis*, we conducted *de novo* transcriptome sequencing which focused on the major feeding stage of fourth-instar larvae, and our work revealed useful information on chitin metabolism and insecticide detoxification and target genes of *C. medinalis*. We acquired 29,367,797 Illumina reads and assembled these reads into 63,174 unigenes with an average length of 753 bp. Among these unigenes, 31,810 were annotated against the National Center for Biotechnology Information non-redundant (NCBI nr) protein database, resulting in 24,246, 8669 and 18,176 assigned to Swiss-Prot, clusters of orthologous group (COG), and gene ontology (GO), respectively. We were able to map 10,043 unigenes into 285 pathways using the Kyoto Encyclopedia of Genes and Genomes Pathway database (KEGG). Specifically, 16 genes, including five chitin deacetylases, two chitin synthases, five chitinases and four other related enzymes, were identified to be putatively involved in chitin biosynthesis and degradation, whereas 360 genes, including cytochrome P450s, glutathione *S*-transferases, esterases, and acetylcholinesterases, were found to be potentially involved in insecticide detoxification or as insecticide targets. The reliability of the transcriptome data was determined by reverse transcription quantitative PCR (RT-qPCR) for the selected genes. Our data serves as a new and valuable sequence resource for genomic studies on *C. medinalis*. The findings should improve our understanding of *C. medinalis* genetics and contribute to management of this important agricultural pest.

## 1. Introduction

The rice leaf folder, *Cnaphalocrocis medinalis* (Guenee) (Lepidoptera: Pyralidae) is one of the most important rice pests in many Asian countries, including Korea, Japan, China, Malaysia, Sri Lanka, and Vietnam [1]. It has five stadiums in larval stage. The larvae can attack all the stages of the rice whereas the adults are capable of successive long-distance nocturnal flights. Starting from the fourth-instar when larvae regularly roll up rice leaves, they become solitary and difficult to control [2]. This insect was considered a minor pest previously, but it appeared to be more important with the spread of high-yield rice varieties and the accompanying changes in cultural practices [3]. The control of rice insect pests often depends on extensive use of chemical insecticides; however, their use has led to poisonings of farmers, degradation of land and water, and increased levels of dangerous chemicals in Chinese food supply [4]. At the same time, the use of synthetic insecticides in crop pest control programs around the world has resulted in disturbance of the environment, pest resurgence, pest resistance to insecticides, and adverse effects on non-target organisms [5]. Hence, it is very urgent to find environmentally friendly approaches to controlling these pests.

In recent years, there are many studies on *C. medinalis* around the world, especially on insecticide resistance. Unfortunately, little is known about the genetic diversity and population structure of *C. medinalis*, which is important to improve our understanding and control of these pests [6,7]. In a recent study, complete mitochondrial genomes (mitogenomes) of *C. medinalis* were determined and analyzed. The circular genomes were 15,388 bp and contained 37 genes [8]. A total of 20 microsatellite markers were isolated and characterized from *C. medinalis* partial genomic libraries using the method of fast isolation by AFLP (amplified fragment length polymorphism) of sequence containing repeats [9]. Transcriptome sequencing for *C. medinalis* was reported in 2012 by Li *et al.* in which a total of 25,281 unigenes matched National Center for Biotechnology Information non-redundant (NCBI nr) protein database and five DGE (digital gene expression profiling) libraries of mixture samples of eggs, 1st to 5th instar larvae, pupae, and adults were constructed [10]. However, a comprehensive description of the whole-genome sequence and the gene families that may be involved in insecticide detoxification and resistance in a particular stage of *C. medinalis* remain unavailable.

Chitin is the second most commonly found amino polysaccharide in nature and mainly synthesized by fungi, nematodes and arthropods. It is a linear polymer of β-(1-4)-linked *N-*acetylglucosamines (GlcNAc) that assemble into microfibrils of varying diameter and length after polymer synthesis and transport to the extracellular space [11]. Chitin widely exists in insect cuticles and peritrophic matrices, where it functions as a permeability barrier between the food bolus and the midgut epithelium, enhances digestive processes and protects the brush border from mechanical disruption as well as from attack by toxins and pathogens [12]. Because chitin also plays an important role during insect molting, it serves as a selective target for pest control [13].

Due to the widespread use of insecticides, resistance to various insecticides in insect populations has become a serious problem. Metabolic resistance and target resistance are the two major types of mechanisms of insecticide resistance. Metabolic resistance relies on detoxification enzymes such as glutathione *S*-transferases (GSTs), carboxylesterases (CarEs) and cytochrome P450 monooxygenases (P450s). Target resistance mainly involves γ-aminobutyric acid (GABA) receptor, acetylcholinesterase (AChE), voltage-gated sodium channel, nicotinic acetylcholine receptor and ryanodine receptor [14,15,16]. GSTs belong to a major family of detoxification enzymes found in insects, including *Drosophila melanogaster* and *Anopheles gambiae*. They can help organisms protect cells from oxidative stress and chemical toxicants by aiding the excretion of electrophilic and lipophilic compounds from the cell. Thus, GSTs are believed to play an important role in the defense of cells against these xenobiotic toxins [17,18]. In previous research, CarEs were identified as a major factor in the metabolism and therefore detoxification of organophosphorus compounds such as soman and trichothecene toxins. In silkworm, comparative genomic analysis suggested that CarEs genes may have important roles in detoxifying secondary metabolites of mulberry leaves, contaminants in diet, and odorants [19,20]. It turned out that P450s played a central role in the adaptation to plant chemicals of insecticide resistance in insect populations [21].

Over the past several years, sequencing of large genomes has driven the search for alternative methods to reduce expenses and increase efficiency [22]. The high-throughput nature of next-generation sequencing technologies now makes it possible to carry out genome-wide studies of transcriptomes in a rapid way and they have been widely used to explore gene structure and expression profiling on model organisms [23]. RNA sequencing represents an attractive alternative to whole-genome sequencing because it only analyzes transcribed portions of the genome, while avoiding non-coding and repetitive sequence that can make up much of the genome [24,25]. Therefore, transcriptome analysis is essential to interpret the functional elements of the genome and reveal the molecular constituents of cells and tissues [26,27].

Fourth-instar larvae of *C. medinalis* often consume large amounts of food and are most destructive to rice. Therefore, we chose the fourth-instar larvae as experimental materials in this study. It has been previously shown that insecticide resistance can be conferred by changed behavior, modified physiological function, enhanced detoxification and reduced target sensitivity [28]. We obtained a series of information of chitin synthesis, degradation related enzymes, and comprehensive information of insecticide detoxification and resistance related genes of *C. medinalis*. Therefore, it will help us to find novel target sites for developing effective, safe and environmentally sound insecticides.

In this study, we focused on the fourth-instar larvae and utilized the Illumina HiSeq™ 2000 sequencing platform and generated 29,367,797 reads of *C. medinalis.* In total, we obtained 63,174 unigenes. All unigenes were annotated to different databases with the aid of bioinformatics tools. Besides, we identified hundreds of insecticide target and metabolism genes, and analyzed putative genes involved in chitin biosynthetic and degradation pathways. Also, a large number of simple sequence repeats (SSRs) were determined. We expect that the new dataset will provide an invaluable resource for identifying important genes for insecticide detoxification and resistance and for development and evolution in *C. medinalis*.

## 2. Results

### 2.1. Illumina Sequencing and Assembly

To obtain a global view of the *C. medinalis* transcriptome and interested genes, total RNA was isolated from the whole bodies of the fourth-instar larvae. We performed high-throughput RNA-Seq (RNA sequencing) experiments using Illumina sequencing technology. According to stringent quality assessment and data filtering, 29,367,797 reads with 97.97% Q20 bases (those with a base quality greater than 20) were produced and the GC content of the transcriptome was 50.36%. To facilitate sequence assembly, these raw reads were randomly clipped into 21-mers for sequence assembly using Short Oligonucleotide Analysis Package (SOAP) *de novo* software [29]. All short-read sequences were assembled into 3,381,765 contigs and 106,407 transcripts with a mean length of 1194 bp. The transcripts were subjected to cluster and assembly analyses and identified 63,174 unigenes (Table S1). The size distribution of contigs, transcripts and unigenes are shown in Figure S1A–C, respectively. In total, contigs that ranged from 0 to 300 bp (98.58%) in size were maximized and 38,440 transcripts (36.13%) were longer than 1000 bp and 19,585 transcripts (18.41%) were longer than 2000 bp. Among these unigenes, 25,837 (40.9%) were longer than 500 bp and 12,724 (20.14%) were longer than 1000 bp.

### 2.2. Open Reading Frame Prediction and Global Gene Expression

We predicted open reading frame (ORF) from the forward and reverse three bases in turn to meet start or end termination codons by using Getorf software [30]. The longest sequence was selected as an ORF for unigene. The length distribution was shown in Figure S1D and Table S2. We analyzed global gene expression at transcript levels by RPKM (the reads per kilo bases per million reads). The expression profiles for 63,174 unigenes (RPKM > 0) were illustrated in Table S3.

### 2.3. Sequence Annotation and Classification

**Figure 1:**
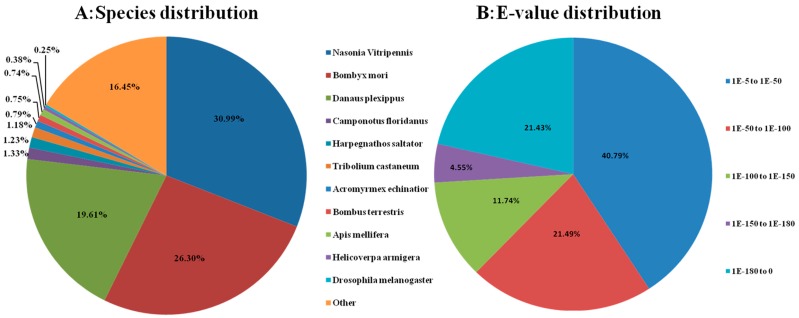
Characteristics of homology search of Illumina sequences against the non-redundant (nr) database. (**A**) Species distribution is shown as the percentage of the total homologous sequences (with an *E*-value ≤1.0 × 10^−5^); (**B**) *E*-value distribution of BLAST hits for each unique sequence with a cut-off *E*-value of 1.0 × 10^−5^. We used all insect proteins in the National Center for Biotechnology Information (NCBI) nr database for homology search and extracted the best hit of each sequence for analysis.

We annotated the assembled sequences by aligning with the deposited ones in various protein databases by using a BLASTX program with a cutoff *E*-value of 10^−5^, including the National Center for Biotechnology Information (NCBI) non-redundant protein (nr) database, the Kyoto Encyclopedia of Genes and Genomes (KEGG), the UniProt/Swiss-Prot, Gene Ontology (GO) and the Cluster of Orthologous Groups of proteins (COG). In total, we annotated 32,035 unigenes to related protein databases (Table 1). Unfortunately, the remaining 31,139 unigenes failed to acquire annotation. The analysis indicated that 31,810 unigenes had significant matches in the Nr database and 24,246 unigenes in the Swiss-Prot database, 32,035 unigenes were successfully annotated in the nr, Swiss-Prot, KEGG, GO, COG database (Table 2). We also predicted some homologous genes from several species, about 30.99% of the unigenes have the highest homology to genes from *Nasonia vitripennis*, followed by *Bombyx mori* (26.30%), *Danaus plexippus* (19.61%), *Camponotus floridanus* (1.33%), *Harpegnathos saltator* (1.23%) and *Tribilium castaneum* (1.18%) (Figure 1A). The *E*-values of the most unique sequences ranged from 1.0 × 10^−5^ to 1.0 × 10^−50^ (Figure 1B).

**Table 1:** Functional annotation of the *C. medinalis* transcriptome.

Annotated Databases	Annotated Number	300 ≤ Length < 1000 bp	Length ≥ 1000 bp
COG_Annotation	8669	3410	4372
GO_Annotation	18,176	7955	7174
KEGG_Annotation	10,043	4217	4482
Swissprot_Annotation	24,246	10,627	10,539
nr_Annotation	31,810	14,722	11,261
All_Annotated	32,035	14,834	11,270

**Table 2:** Summary of simple sequence repeat (SSR) types in the *C. medinalis* transcriptome.

Repeat Motif	Number	Percentage (%)
Single-nucleotide		
A/T	4803	
C/G	247	
Total	5050	48.59
Di-nucleotide		
AC/GT	372	
AG/CT	775	
AT/AT	535	
CG/CG	144	
Total	1826	17.57
Tri-nucleotide		
AAC/GTT/AAG/CTT/AAT/ATT	757	
ACC/GGT/ACG/CGT/ACT/AGT	800	
AGC/CTG/AGG/CCT/ATC/ATG/CCG/CGG	1851	
Total	3408	36.60
Tetra-nucleotide		
AAAC/GTTT/AAAG/CTTT/AAAT/ATTT/AACC/GGTT	54	
AACG/CGTT/AAGC/CTTG/AAGG/CCTT/AATC/ATTG	11	
AATG/ATTC/ACAG/CTGT/ACAT/ATGT/ACCG/CGGT	13	
ACCT/AGGT/ACGC/CGTG/ACGG/CCGT/ACTC/AGTG	10	
ACTG/AGTC/AGAAGCG/CGCTT/ATCT/AGCC/CTGG/	6	
AGGC/CCTG/AGGG/CCCT/ATCC/ATCG/ATCG/ATCG	7	
Total	101	0.97
Penta-nucleotide		
AAAAC/GTTTT/AAAAT/ATTTT	3	
AAGTG/ACTTC/AATTC/AATTG	2	
AGGCG/CCTCG	1	
Total	6	0.058
Hexa- nucleotide		
ACCATG/ATGGTC	1	
ACCGCC/CGGTGG	1	
AGCCGC/CGGCTG	1	
Total	3	0.029

The A/T/C/G motifs were the most abundant repeat type, and accounted for approximately half of the total number of SSRs. In previous research, 20 microsatellite markers were isolated and characterized from *C. medinalis* using the method of fast isolation by Amplified fragment length polymorphism (AFLP) of sequence containing repeats. According to our transcriptome sequencing databases, we obtained a large number of SSRs. The result would facilitate the future study on population genetics and molecular genetics of *C. medinalis* and would also be useful for species taxonomy study considering the migratory character of *C. medinalis*.

**Figure 2:**
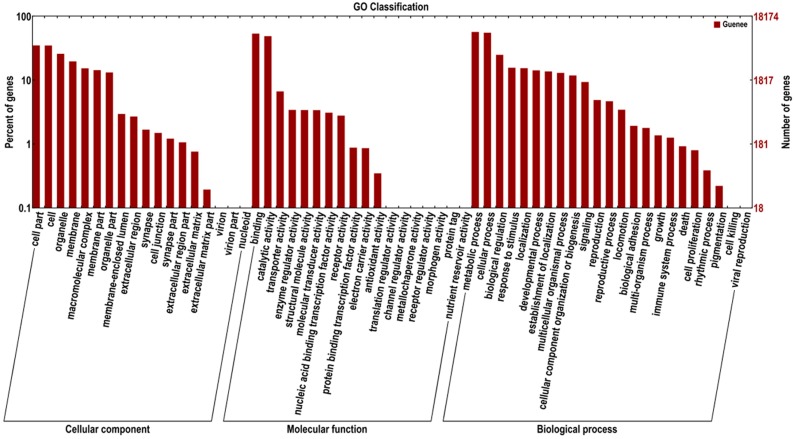
Functional annotation of assembled sequences based on gene ontology (GO) categorization. GO analysis was performed at the level two for three main categories (cellular component, molecular function, and biological process).

GO assignments were used to illustrate the functions of the unigenes. In each of the three main categories (biological process, cellular component and molecular function) of the GO classification, 18,176 unigenes were annotated. They were divided into three ontologies: 28,634 unigenes for molecular function, 14,315 for cellular components, and 36,931 for biological processes. For biological processes, genes involved in metabolic process, cellular process and biological regulation were highly represented. For molecular functions, binding (9729) and catalytic activity (8882) were the most represented GO term, followed by transporter activity (1210). Regarding cellular components, the most represented category was cell part (6367) and cells (6339) (Figure 2).

**Figure 3:**
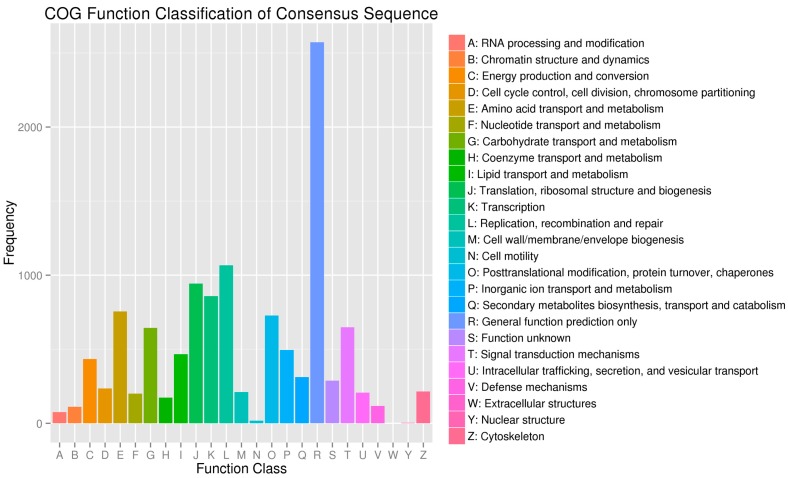
Clusters of orthologous group (COG) classification. A total of 8669 unigenes with non-redundant database hits were grouped into 25 COG classifications.

In addition, all unigenes were subjected to a search against the COG database for functional prediction and classification. Every protein in the COG database was assumed to be evolved from an ancestor, and the whole database was built on coding proteins with complete genomes as well as system evolution relationships of bacteria, algae and eukaryotes. In total, 8669 of the 63,174 (13.72%) unigenes with hits in the nr database could be assigned to COG classification and divided into 25 specific categories (Figure 3). COG-annotated putative proteins were functionally classified into at least 25 protein families involved in cellular structure, biochemistry metabolism, molecular processing, signal transduction and other functions. The cluster for general function prediction (2572; 29.67%) represented the largest group, followed by replication, recombination and repair (1066; 12.30%), translation, ribosomal structure and biogenesis (943; 10.88%), transcription (858; 9.90%), amino acid transport and metabolism (755; 8.71%), posttranslational modification, protein turnover, chaperones (727; 8.39%), signal transduction mechanisms (649; 7.49%), carbohydrate transport and metabolism (644; 7.43%), inorganic ion transport and metabolism (494; 5.70%), lipid transport and metabolism (466; 5.38%), and energy production and conversion (434; 5.0%). Only a few unigenes were assigned to nuclear structure (4) and cell motility (17).

The metabolic pathways of the unigenes were also analyzed using the KEGG annotation system. This process predicted a total of 285 pathways, which represented a total of 10,043 unigenes. The pathways involving the highest number of unique transcripts were metabolism (26.10%), followed by RNA transport (4.32%) and spliceosome (3.61%) (Table S4).

### 2.4. Simple Sequence Repeats (SSR) Discovery

Simple sequence repeats (SSRs) are ubiquitous in eukaryotes and widely used for genetics and evolution studies. It has been reported that approximately 3%–7% of expressed genes contain putative SSR motifs, mainly within the un-translated regions of the mRNA. To further evaluate the assembly quality and develop new molecular markers of *C. medinalis*, the 12,724 unigene sequences were submitted to an online service to search for SSRs. In total, 10,394 SSRs were detected, including 5050 (48.59%) single nucleotide motifs, 1826 (17.57%) dinucleotide motifs, 3408 (36.60%) trinucleotide motifs, 101 (0.97%) tetranucleotide motifs, six pentanucleotide motifs and three hexanucleotide motifs (Table 2).

### 2.5. Transcripts Encoding Chitin Metabolism Enzymes

**Figure 4:**
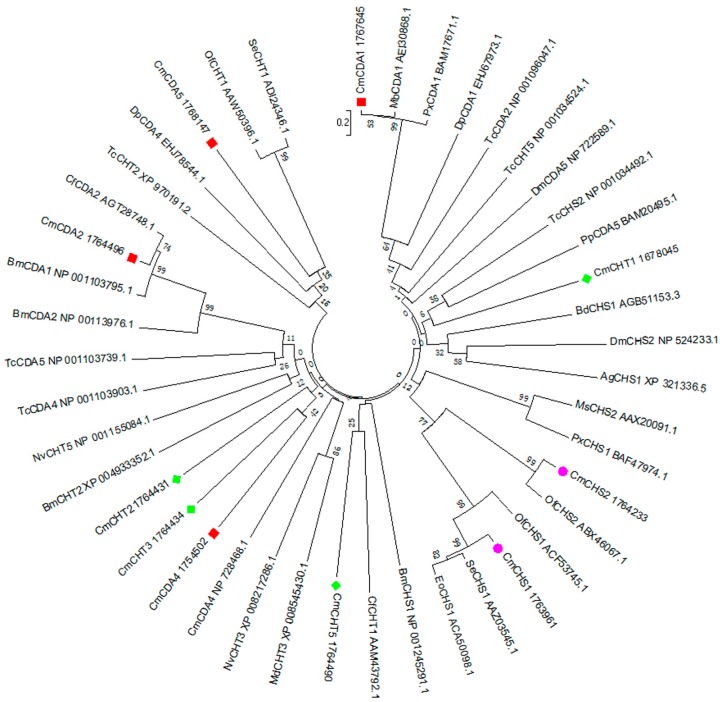
Phylogenetic relationships of chitin-related enzymes deduced from the open reading frame (ORF) among *C. medinalis* and other insect species. The tree was constructed from the multiple alignments using MEGA 6.0 program (1000 bootstrap replications). Bootstrap values >50% are shown. The *C. medinalis* enzymes are indicated by red, green or pink markers. Cm, *Cnaphalocrocis medinalis*; Bm, *Bombyx mori*; Of, *Ostrinia furnacalis*; Px, *Plutella xylostella*; Se, *Spodoptera exiqua*; Eo, *Ectropis oblique*; Ms, *Manduca sexta*; Ag, *Anopheles gambiae*; Dm, *Drosophila melanogaster*; Bd, *Bactrocera dorsalis*; Tc, *Tribolium castaneum*; Mb, *Mamestra brassicae*; Dp, *Danaus plexippus*; Cf, *Choristoneura fumiferana*; Nv, *Nasonia vitripennis*; Md, *Microplitis demolitor*; Px, *Papilio xuthus*; Pp, *Papilio polytes.*

Insect growth and development consistently synthesize and degrade chitin in a highly controlled manner to allow ecdysis and regeneration of the peritrophic matrices. Chemical compounds that interfere with chitin metabolism, such as diflubenzuron, have been of special interest for the control of agricultural pests. In our annotated *C. medinalis* transcriptome dataset, 16 genes encoding chitin enzymes which were involved in main chitin biosynthesis and degradation were identified. These genes putatively encode chitin deacetylases (CDA1, 2, 4, 5, 6), chitin synthase 1 (CHS1), chitin synthase 2 (CHS2), chitinases (CHT1, 2, 3, 5, 7), *N*-acetyl-d-glucosamine kinase (NAGK), *N-*phosphoacetylglucosamine mutase (PAGM), UDP-*N*-acetylglucosamine pyrophosphorylase (UAP) and β-l-*N*-acetyhexosaminidase (Hex) (Table 4). Among these genes, the full-length cDNA sequences of nine genes were obtained by transcriptome datasets combined with rapid-amplification of cDNA ends (RACE) and were submitted to NCBI (Table 3). The phylogenetic tree of the major chitin metabolic enzymes including CDAs, CHTs and CHSs deduced from the ORFs in 17 insect species were constructed using MEGA 6.0 (Figure 4). A phylogenetic showed that *C. medinalis* chitin synthase 1 (*CmCHS1*) had the closest relationship with *Spodoptera exiqua* chitin synthase 1 (*SeCHS1*), *CmCHS1* have the closest relationship with *Ostrinia furnacalis* chitin synthase 1 (*OfCHS1*). Additionally, the *C. medinalis* chitin deacetylase 1 (*CmCDA1*) seem to have the closest relationship with *Mamestra brassicae* chitin deacetylase1 (*MbCDA1*), and *CmCDA2* keep closest relationship with *Choristoneura fumiferana* chitin deacetylase 2 (*CfCDA2*). These results suggested their similar physiological functions and evolutionary relatedness with those of other insect species.

**Table 3:** The major enzyme genes involved in chitin metabolism in *C. medinalis*.

Gene Name	GeneBank Accession No	Length (bp)	Function (Ontology)
Chitin synthase 1 (CHS1)	KP000843	4868	chitin biosynthetic process
Chitin synthase 2 (CHS2)	KP000844	4651	
*N-*Phosphoacetylglucosamine mutase (PAGM)	KP000845	1934	carbohydrate metabolic process phosphoacetylglucosamine mutase activity, UDP-*N*-acetylglucosamine biosynthetic process
UDP-*N*-acetylglucosamine pyrophosphorylase	KP000846	1173 (partial)	transferase activity, transferring glycosyl groups, UMP salvage catalytic activity
*N*-Acetyl-d-glucosamine kinase(NAGK)	KP000849	733 (partial)	*N*-acetylglucosamine kinase activity
Chitinase 1	KF897513	2039	chitin catabolic process, carbohydrate metabolic process, chitinase activity chitin binding, carbohydrate metabolic process, extracellular region, cuticle chitin catabolic process, chitinase activity chitin catabolic process, carbohydrate metabolic process, imaginal disc development, extracellular region, chitinase activity chitin catabolic process, carbohydrate metabolic process, chitinase activity β-*N*-acetylhexosaminidase activity peptidoglycan-based cell wall biogenesis peptidoglycan turnover, chitin catabolic process, chitin deacetylase activity
Chitinase 2	KP000847	1897	
Chitinase 3	KP000848	4311	
Chitinase 5	KP000850	2105 (partial)	
Chitinase 7	none	none	
β-l-*N*-Acetylhexosaminidase	KP000851	1377 (partial)	
Chitin deacetylase 1	KP000854	2020	
Chitin deacetylase 2	KP000852	2158	
Chitin deacetylase 4	KP000853	1537	
Chitin deacetylase 5	1768147	765 (partial)	
Chitin deacetylase 6	1787955	349 (partial)	

**Table 4:** Primers used in sequencing the full-length cDNA and reverse transcription quantitative PCR (RT-qPCR) analysis.

Gene Name	Primer Sequence	Length of Product (bp)
**Reverse Transcription Quantitative PCR (RT-qPCR)**		
*CmCHS1*	F: CTGGAACAGCAACGGAAACT	190
	R: TCCGTTGTTGATGAGCCAGA	
*CmCHT1*	F: TGCTGGCAACAACAACTACAATC	231
	R: ATCTCCACGCTCTTAGGGTCTT	
*CmCDA1*	F: AAGAAATGGCGGGTATGAGGGTG	142
	R: AGTGGTGGATTAGACAAAGGTGCG	
*CmActin*	F: GAGCGTGGTTACTCATTCA	283
	R: TGTCAACATCGCACTTCA	
*CmCYPA792*	F: TTGATGCGGATGTTGACG	164
	R: ATGCCCTTTGGAGTTGGA	
*CmGST*	F: AACTGTATTCGGCTTTATCC	234
	R: ACACACTTGCTGCCTTTTCC	
**PCR for cDNA Sequencing**		
*CmCDA1*	F1: GTCTACGGTTGCGTTGCTCC	911
	R1: GAGCAACGCAACCGTAGACGACTGG	
	F2: CATCATCGTTGTGCGTGACAGAGTG	970
	R2: ACGACAGCACAATCACCGCACCTT	
	F3: GCCGTCCTCGGAGCAGAAACAGTCA	830
	R3: CCAACAACGA CGAATATCTTCCAGG	
*CmCHT1*	F1: TGTCCTCGATGCCGTACCTGCCCAC	1300
	R1: AAGGGGTGCTGACCGCTGCTGTGCCGCT	
	F2: CACGCCGGGGCGGTACACCGCC	300
	R2: TGGGAAGACAAGGGCTGTCCAACCAACA	
*CmCHSA*	F1: ACTATGTGGCACGAAACGAA	354
	R1: TAGTACATGTACAT	
	F2: CATTTGAAAGATAAGGC	580
	R2: ACCAACATRAGRAADAT	
	F3: CGCCTTACATCGCTTACC	1258
	R3: ACBARACCRATNGGYTCC	

### 2.6. Reverse Transcription Quantitative PCR (RT-qPCR) Analysis

**Figure 5:**
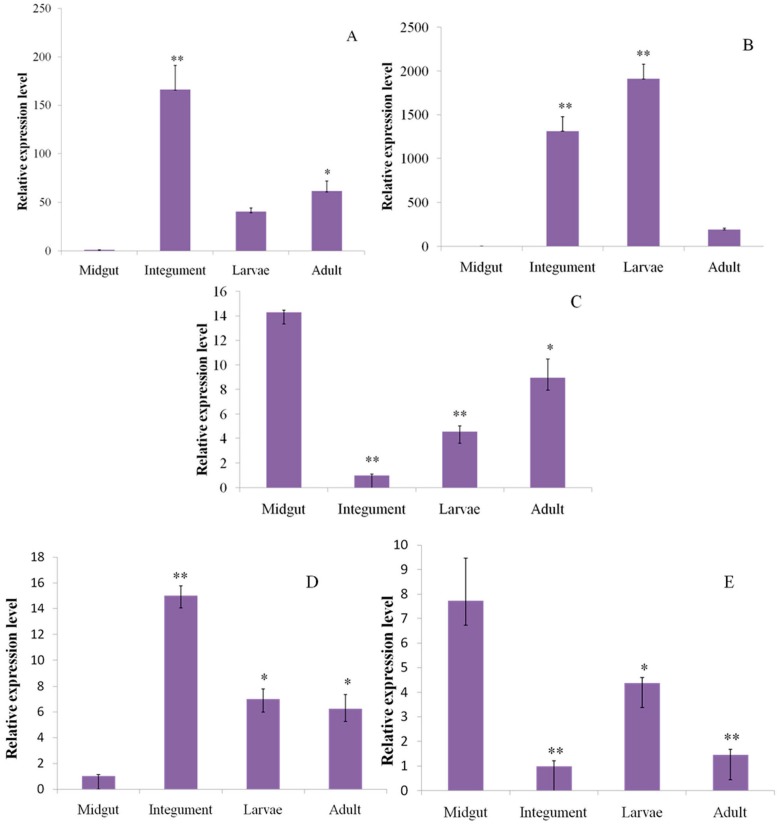
Real-time PCR analysis of the transcripts putatively encoding chitin metabolism enzymes and detoxification-related enzymes in *C. medinalis*. (**A** to **E** refer to relative expression level of *C. medinalis* chitin synthase 1 (*CmCHS1*), chitinase 1 (*CmCHT1*), chitin deacetylase 1 (*CmCDA1*), cytochrome P450 monooxygenase CYP9A79 (*CmCYP9A79*) and glutathionine *S*-transferase (*CmGST*). * *p* < 0.05, ** *p* < 0.01).

In order to determine the reliability of the transcriptome, the main genes involved in chitin metabolism, their relative expression levels were determined by RT-qPCR analysis in two tissues (integument and midgut) and two developmental stages (larvae and adult) (Figure 5). The relative quantification showed significantly higher level of expression of *CmCHS1* in the integument than other tissues or developmental stages (Figure 5A). *CmCHT1* showed high expression in the larvae and integument, but low expression in the midgut and adults (Figure 5B). In contrast, *CmCDA1* showed higher expression in the midgut (Figure 5C).

### 2.7. Chitin Metabolism Enzyme Networks of C. medinalis

**Figure 6:**
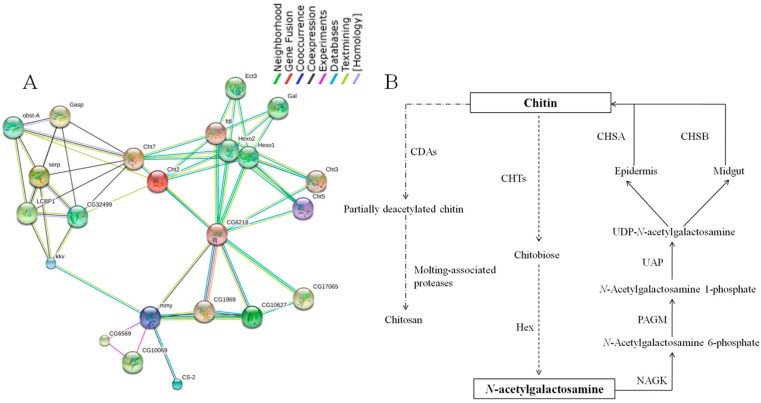
(**A**) Interaction networks of chitin enzymes associated with chitin biosynthesis based on the STRING website (focused on a specific protein network in *Drosophila melanogaster*); (**B**) Chitin metabolic pathway and related genes in *C. medinalis*. The solid arrow indicates chitin biosynthetic pathway; The dotted arrow and chain line arrow indicates chitin degradation pathway; CDA: chitin deacetylase, CHT: chitinase, Hex: β-l-*N*-acetylhexosaminidase, CHS: chitin synthase, UAP: UDP-*N*-acetylglucosamine pyrophosphorylase, PAGM: phosphoacetylglucosamine mutase, NAGK: *N*-acetyl-d-glucosamine kinase.

The functional association networks of the 16 chitin metabolism enzymes were generated using STRING 9.1 (http://string-db.org/). Since proteins are often involved in multiple cellular processes or metabolic pathways, these close interaction patterns, evident with high confidence score, are helpful for a better understanding of the activities of each individual protein when observed in a network of associations [31]. As illustrated in Figure 6A, the highly conserved protein chitin synthase 1 (krotzkopf verkehrt, kkv) had high score (>0.9) with coexpression evidenced close relation to chitin deacetylase family CDA1 (serp), CDA2 (LCBP1), CDA4 (CG32499) and several other functional partners like obstA and Gasp which has chitin binding domain. Chitin deacetylase family has the function of catalyzing the deacetylation of chitin [32]. Chitin synthase 1 (kkv) also showed a close relationship with UDP-*N*-acetylglucosamine pyrophosphorylase (UAP, mmy). *N*-Acetyl-d-glucosamine kinase (NAGK, CG6218) seems to be the center to connect the family of chitinase (Cht), β-l-*N*-acetylhexosaminidase (Hex) and phosphoacetylglucosamine mutase (PAGM, CG10627) then to mmy. But for chitin synthase 2 (cs-2), there is only connection to mmy. According to the protein interaction network, we obtained a model of chitin metabolic pathway in *C. medinalis* (Figure 6B). Chitin formation can be divided into three simplified steps. In the first step, *N*-acetylgalactosamine (GlNAc) is phosphorylated by enzymes NAGK to form *N*-acetylglucosamine 6-phosphate. The second step, UDP-*N*-acetylglucosamine (UDP-GlcNAc) is the active form of *N*-acetylglucosamine (GlcNAc) and its formation is catalyzed by UDP-GlcNAc pyrophosphorylase (UAP) in the cell cytoplasm [33]. UAP is also important for glycosylation of proteins, sphingolipids and secondary metabolites with GlcNAc or glycosyl-phosphatidyl inositol (GPI) anchors which bridge the proteins to cell membrane, or for conjugation of 7-β-hydroxylated bile acids [34,35]. The third step completes the process: UDP-GlcNAc is added to the growing chitin polymer by the membrane-integrated enzyme chitin synthase. The polymerization requires UDP-GlcNAc as a substrate and divalent cations as co-factors [36].

### 2.8. Identification of Insecticide Resistance Related Genes and RT-qPCR Validation

By using BLASTX, we identified a series of transcripts (Table S5) related to insecticide detoxification, including cytochrome P450, carboxylesterase, glutathione *S*-transferases, and insecticide target proteins, including acetylcholinesterase, γ-aminobutyric acid receptor, nicotinic acetylcholine receptor and sodium channel [10]*.* In total, 360 transcripts were identified in relation to insecticide detoxification or as targets, which were divided into 12 groups: cytochrome P450, carboxylesterase, glutathione *S*-transferases, GABA receptor, nicotinic acetylcholine receptor, sodium channel, acetycholinesterases, aquaporin, calcium channel, chloride channel, methoprene-tolerant, and serine protease inhibitor. The largest and most abundant group was cytochrome P450s (129, 35.83%) followed by serine protease inhibitors (41, 11.39%). Furthermore, we also analyzed the major subclasses of glutathione *S*-transferases and carboxylesterases. In order to determine the reliability of the transcriptome, we selected two detoxification genes to perform qRT-PCR validation, which included *C. medinalis* cytochrome P450 monooxygenase CYP9A79 (*CmCYP9A79*) and glutathionine *S*-transferase (*CmGST*)*.* Our results showed that *CmCYP9A79* was differentially expressed in the integument, midgut, larvae and adults. The expression levels of *CmCYP9A79* were similar in larvae and adults. However, its expression was significantly higher in the integument than in the midgut (Figure 5D). In contrast, *CmGST* showed a higher expression level in the midgut than in the integument (Figure 5E).

## 3. Experimental Section

### 3.1. Insect Sample Preparation

The *C. medinalis* larvae were collected from the paddy fields in Rice Research Institute of Anhui Academy of Agricultural Sciences. The well-developed larvae of the fourth instar were used in the study. All samples were washed with diethyl pyrocarbonate (DEPC)-treated water, immediately frozen in liquid nitrogen and stored at −80 °C until use.

### 3.2. Isolation of Total RNA and cDNA Library Construction

Total RNA was extracted from the mixture of 10 whole larvae of *C. medinalis* using TRIzol reagent (Invitrogen, Grand Island, NY, USA). RNA was quantified by measuring the absorbance at 260 nm using a NanoDrop2000 spectrophotometer (Thermo Fisher Scientific, New York, NY, USA). The purity of all RNA samples were assessed at an absorbance ratio of A_260/280_ and A_260/230_, and the integrity of RNA was confirmed by 1.8% agarose gel electrophoresis. Sequencing was completed using an Illumina genome analyzer. Briefly, mRNA was purified from RNA using oligo (dT) magnetic beads and fragmented. Total first-strand cDNA was generated using random hexamer-primed reverse transcription, followed by synthesis of the second-strand cDNA using RNase H and DNA polymerase I. Then, single run paired-end RNA-Seq libraries were prepared following Illumina’s protocols and sequenced on the Illumina sequencing platform.

### 3.3. Sequence Data Analysis and Assembly

The raw reads generated by Illumina HiSeq™ 2000 (Illumina, San Diego, CA, USA) were initially processed to obtain high-quality clean reads. Then, all the clean reads were assembled using a *de novo* assembly program using Trinty and SOAP *de novo*, which recovers more full-length transcripts across a broad range of expression levels, with sensitivity similar to methods that rely on genome alignments. Trinity is an efficient *de novo* transcriptome assembler in the absence of a reference genome. It combined all reads with a certain overlap length to from contigs, reads were then mapped back to contigs. These contigs were connected into sequences that could not be extended on either end, which were defined as unigenes [37].

### 3.4. Functional Annotation and Classification

The optimal assembly results were chosen following the assembly evaluation. The clustering analysis was performed to achieve a unigene database that was comprised of the potential alternative splicing transcripts. The assembled sequences were compared against the NCBI nr and non-redundant nucleotide sequence (Nt) database, and Swiss-Prot database using Blastn with an *E-*value *<*10^−5^. To annotate the assembled sequences with GO terms, the Swiss-Prot Blast results were imported into Blast2GO, a software package that retrieves GO terms, allowing gene functions to be determined and compared. The unigene sequences were also aligned to the COG database to predict and classify functions. Then, KEGG pathways were assigned to the assembled sequences using the online KEGG Automatic Annotation Sever (KAAS). Open reading frames (ORFs) were predicted using the Getorf software [38]. We quantified expression levels in reads per kilobase of exon model per million mapped reads (RPKM). The RPKM measure of read density reflects the molar concentration of a transcript in the starting sample by normalizing for RNA length and for the total read number in the measurement [39]. Finally, the best matches were used to identify coding regions and determine the sequence direction.

### 3.5. Identification of Insecticide Resistance Genes and Chitin Metabolism Enzymes

Enzymes involved in chitin metabolism and genes related to insecticide resistance were identified using *C. medinalis* unigenes. They were searched by BLASTN with an *E-*value threshold of 1 ×10^−5^. If no hits were produced from BLASTN alignments, those nucleotide sequences were downloaded for TBLASTX alignments (*E-*value threshold: 1 × 10^−5^). We compared the representative transcriptome to a custom BLAST database containing insecticide-related genes from *Bombyx mori*, *Danaus plexippus*, *Nasonia vitripennis*, *Spodoptera littoralis*, *Manduca sexta*, *Helicoverpa armigera* and *Papilio xuthus et al.* The complete coding region was determined using the open reading frame (ORF) finder and protein BLAST results. The phylogenetic tree was constructed by MEGA 6.0 software from different insect species [40].

### 3.6. SSR Detection

The 63,174 unigenes of *C. medinalis* obtained in the present study were also subjected to the detection of SSRs using the online program of the Simple Sequence Repeat Identification Tool [41]. The parameters were adjusted for identification of perfect di-, tri-, tetra-, penta- and hexa-nucleotide motifs with a minimum of 6, 5, 5, 5 and 5 repeats, respectively. The report of this search included the total number of sequences containing SSRs among the submitted unigenes, sequence ID, SSR motifs, repeats numbers, repeat length, SSR starts and SSR ends.

### 3.7. The Predicted Functional Association Networks

The STRING database (version 9.1, http://string-db.org/) was used to generate high-confidence protein-protein interaction networks that demonstrate direct interactions of these proteins with other associated proteins. Due to the lack of proteomic information of *C. medinalis*, the protein–protein interaction (PPI) networks of chitin metabolism related proteins were generated using the database of another well-studied insect, *D. melanogaster.*

### 3.8. cDNA Cloning of Chitin Genes and Sequence Analysis

Total RNA was prepared from the fourth-instar larvae using TRIzol^®^ Reagent (Invitrogen). For reverse transcription, total RNA was treated with RQ1 RNasefree DNase I (Promega, Madison, WI, USA) at 37 °C for 30 min to remove contaminated genomic DNA, and DNase was inactivated by heating to 65 °C for 20 min. Reverse transcription was performed using oligo (dT) primer (Promega) and ImProm-II reverse transcriptase (Promega) following the manufacturer’s instructions. The full length cDNA of the chitin metabolic genes were sequenced using the specific primer (Table 4). The 5′ and 3′ RACE were performed using smarter race kit (Clontech, Mountain View, CA, USA). The ORFs were predicted from the nucleotide sequence using DNAMAN (Lynnon Biosoft, Vandreuil, QC, Canada). BLASTP (http://www.ncbi.nlm.nih.gov/) was used to search homologous and protein sequences of these chitin genes. Multiple sequence alignment was performed by ClustalW2 with protein sequences retrieved from the NCBI database using default settings, and phylogenetic tree was generated from the conserved regions of 16 proteins by neighbor-joining method with bootstrap of 1000 replications using MEGA 6.0 [42]. The functions annotations of the gene functions were carried out using gene ontology online website (http://geneontology.org/).

### 3.9. RT-qPCR Analysis

In order to validate the results of transcript abundances from our transcriptome analysis, the relative expression levels of three selected genes were analyzed in different tissues and developmental stages by RT-qPCR. Total RNAs were extracted from midgut, integument, larvae and adult using TRIzol reagent. The midgut and integument tissues were dissected from fourth-instar larvae of *C. medinalis*. The concentration of each RNA sample was adjusted to 1 µg/µL with nuclease-free water and total RNA was reverse transcribed in a 20 µL reaction system using the PrimeScript™ RT Reagent Kit with gDNA Eraser (TaKaRa, Dalian, China). The RT-qPCR was carried out in a 25 µL reaction mixture containing 12.5 µL of SYBR Premix Ex Taq (TaKaRa). PCR amplification was performed in triplicate wells. The *C. medinalis* actin (*CmActin*) was used as a reference gene. The thermal cycling profile consisted of initial denaturation at 95 °C for 30 s and 40 cycles of 95 °C for 5 s, 55 °C for 30 s, and 72 °C for 20 s. The reactions were performed in 96-well plates with a Multicolor Real-time PCR Detection System (Bio-Rad, Hercules, CA, USA). Relative expression levels were calculated using the 2^−ΔΔ^*^C^*^t^ method following the previously published protocol [43]. All primer efficiencies were determined according to making standard curves. The qPCR primer efficiencies were 98.52%, 97.24%, 95.71%, 94.13%, 95.48% and 96.20% by the formula of 10^−1/slope^ − 1. Primers used in RT-qPCR were shown in Table 4.

## 4. Discussion

In many Asian countries, *C. medinalis* (Guenee) is one of the most important rice pests. Its outbreak often causes severe yield losses and changes in cropping system. Therefore, it is urgent to find a novel approach to controlling this rice pest. Although the transcriptome sequencing has been applied to *C. medinalis*, there was a lack of information on the genes related to insecticide detoxification, insecticide resistance and chitin metabolism.

In the present study, we chose the fourth-instar larvae as experimental material and applied Illumina paired-end sequencing technology to execute the whole transcriptome of *C. medinalis* in order to focus on the information of the larva. Our database has approximately 29.3 million reads and was assembled into 63,174 unigenes. In contrast, a report by Li *et al.* annotated 25,281 unigenes, and 19,210 unigenes failed to acquire annotation information in the Nr database. Thus, the current number of unigenes was approximately 1.4-fold higher than a previous prepared dataset. As a result, 32,035 unigenes (50.71% of all assembled unigenes) returned significant hits from BLAST comparisons with the six databases. The functional annotation is mainly derived from GO, COG and KEGG databases.

Using KEGG pathway annotation, the genes associated with chitin synthesis and degradation were identified. Chitin widely exists in insect cuticles and peritrophic matrices and plays very important function in insect development. Based on the result of transcriptome sequencing, we analyzed the genes of chitin biosynthetic and degradation pathways in *C. medinalis*. Our analysis revealed 16 enzymes belonging to seven categories: *N-*acetylglucosamine kinase (EC2.7.1.59), phosphoacetylglucosamine mutase (EC5.4.2.3), UDP-*N-*acetylglucosamine diphosphorylase (EC2.7.7.23), chitin synthase (EC2.4.1.16), chitinase (EC3.2.1.14), β-l-*N-*acetylhexosaminidase (EC3.2.1.52), and chitin deacetylase (EC3.5.1.41). During insect development, coordination of chitin synthesis and its degradation requires strict control of the participating enzymes. The chitinases: CHT1, CHT2, CHT3, CHT5 and CHT7 were identified as a family in our transcriptome database. These chitinases belong to family of 18 glycosylhydrolases that hydrolyze chitin by an endo-type of cleavage while retaining the anomeric β-(1,4) configuration of products. There are multiple genes encoding chitinases and chitinase-like proteins in all insect species which differ in size, domain organization, physical, chemical and enzymatic properties, and in patterns of their expression during development, and in tissue specificity of expression [44]. Some RNA interference experiments demonstrate that at least some of these chitinases belonging to different groups serve non-redundant functions and are essential for insect survival, molting or development [45]. Chitinases also show important roles in development and formation in evolution process of *C. medinalis*. Two chitin synthase genes (*CHS1* and *CHS2*, also referred to as *CHSA* and *CHSB*, respectively), have been reported in insects. *CHS1* is responsible for chitin synthesis in cuticle and cuticular lining of the foregut, hindgut, and trachea, whereas *CHS2* is dedicated to chitin synthesis in the PM (peritrophic matrix) [46]. They belong to the large family of glycosyltransferase, a ubiquitous group of enzymes that catalyze the transfer of sugar moieties from activated sugar donors to specific acceptors, thereby forming a glycosidic bond [47], and are large transmembrane proteins with slightly acidic isoelectric points.

According to the network of *D. melanogaster* (Figure 5A), CHS1 connects to chitin deacetylase, whereas CHS2 has different function. CHS2 relates to Mhc1 (myosin heavy chain-like protein) [48], Hil (Hillarin protein), dgt3 (dim γ-tubulin 3) and Pten (actin binding, actin filament organizing). These proteins have actin binding functions, or may participate in actin cytoskeleton organization. Chitin deacetylases (CDAs, EC3.5.1.41) are secreted proteins belonging to a family of extracellular chitin-modifying enzymes that deacetylate chitin to form chitosan, a polymer of β-(1,4)-linked d-glucosamine residues. Nine genes encoding CDA-like proteins were identified in *Tribolium*. Most of the CDAs have a putative signal peptide consistent with their role in modifying extracellular chitin in both cuticle and peritrophic matrix during morphogenesis and molting. Comparative analysis of CDA families in other insect species including *D. melanogaster*, *A. gambiae* and *Apis mellifera*, indicated that the number of CDA genes varies from species to species [49]. Phylogenetic analysis showed that insect CDAs were clustered into five major groups. Group I, III and IV CDAs are found in all 15 insect species [50].

In order to confirm the result of the transcriptome data, we chose three genes for RT-qPCR analysis, including *CmCHS1*, *CmCHT1* and *CmCDA1*. The result showed that *CmCHS1* had a high expression level in the integument. The presence of chitin in the integument has been well documented in insects, which may imply their roles in keeping insect structure and defensed outside microorganism. In recent research, two chitin synthase genes were identified in *A. gambiae*. Transcriptional analysis indicated that *AgCHS1* was expressed in larvae and adults by RT-PCR [51]. In addition, we detected the expression of *CmCDA1* in different tissues; a relatively high level of *CmCDA1* was found in the midgut. However, it is unknown as to why the expression of *CmCDA1* is higher in the midgut. Furthermore, we also detected *CmCHT1* in different tissues and different developmental stages, the result showed high expression of *CmCHT1* in larvae and the integument. Interestingly, we found high expression of the three major genes in larval tissue (Figure 5A–C), hence, it revealed that three major genes were existent and indicated the reliability of our transcriptome data. This is the first report on *C. medinalis* CDAs; further understanding of chitin metabolic pathways will support the development of interventions to selectively manage agriculturally important insect pests.

In past years, the effect of botanical insecticides and bacterial toxins on gut enzyme activity of *C. medinalis* larvae has been investigated. Gut enzyme activities were affected by botanical and bacterial toxin individually and in combination. The intoxication of an insect by an insecticide encompasses three levels of pharmacokinetic interactions: penetration of barrier tissues, distribution and storage, and metabolism in internal tissues [52,53]. Many studies show that insecticides are a pivotal component in controlling *C. medinalis* populations throughout the world [54,55]. Therefore, it is essential to develop potent insecticides against a wide variety of molecular targets in *C. medinalis*. In our transcriptome data, we identified a series of genes related to insecticide detoxification and targets by using the BLASTX program and referring to relevant references. The CYP9A P450 family is an important family related to the detoxification of xenobiotics. We found *CmCYP9A76* was highly expressed in the integument, a well-known structure for the first barrier in defense against outside microorganisms. The detoxification series include P450s, CarEs and GSTs, which are three major families that are primarily responsible for xenobiotic metabolism. The cytochrome P450s expressed in these tissues were expected to be closely related to the metabolism of xenobiotics. The result showed that cytochrome P450s and serine protease inhibitors had highest numbers among the gene families analyzed in this study. Cytochrome P450s are involved in many cases of insecticide resistance since they contribute to xenobiotics. Two counter defensive allelochemical-metabolizing cytochrome P450s including CYP6B1 and CYP6B8 have been identified from *Papilio polyxense* and *Helicoverpa zea* [56,57]. We speculated that many of the cytochrome P450s that we identified in this study could be involved in detoxifications xenobiotics.

It has been reported that serine protease inhibitors play a potent defensive role against predators and pathogens in the plant [58]. In the last decade, studies of serine protease inhibitors have been focused on the functions in controlling the target proteins in a variety of fundamental proteolytic processes in invertebrates such as insects (immune system, digestion, protection against their predators) [59]. Since *C. medinalis* is a migratory insect, we suspect that serine protease inhibitors will play an important role against predators and other microorganisms. Furthermore, we also analyzed the six major subclasses of GSTs: ς, ω, θ, ζ, δ and ε. A total of 39 GSTs-related transcripts were found in our transcriptome data. GSTs are a diverse family of enzymes found ubiquitously in aerobic organisms. These enzymes play a central role in the detoxification of both endogenous and xenobiotic compounds and are also involved in intracellular transport, biosynthesis of hormones and protection against oxidative stress. GSTs can mediate resistance to organophosphate (OP), organochlorines and pyrethroid insecticides [60,61]. We found *CmGST* was highly expressed in the midgut. Insect midgut is regarded as a major tissue for metabolizing various chemicals from ingested food. The high expression of *CmGST* suggested that this gene could be involved in the metabolism of xenobiotics.

Among our gene groups that could potentially contribute to insecticide detoxification or serve as insecticide targets, eight groups have not been previously reported in *C. medinalis*. These groups include aquaporin, calcium channel, chloride channel, methoprene-tolerant, and serine protease inhibitors. In the citrus psyllid, many of the gene groups (e.g., aquaporin and serine proteases) are considered to be relevant to insecticide targets, detoxification and resistance [54]. Aquaporins are integral membrane channel proteins that facilitate the bidirectional transfer of water or other small solutes across biological membranes involved in numerous essential physiological processes [62]. Serine proteases regulate several invertebrate defense response, including hemolymph coagulation, antimicrobial peptide synthesis, and melanization of pathogen surfaces. According to comparative proteomic analysis, serine proteases play a critical role in silkworm resistance against *Bombyx mori* nuclear polyhedrosis virus (BmNPV) infection [63,64].

We also found many simple sequence repeats (SSRs) that are highly informative and widely used for evolution, breeding and genetics studies in *C. medinalis*. The SSRs are useful as molecular markers because their development is inexpensive [65,66].

Overall, our data significantly improves our genetic understanding of *C. medinalis* and provides a substantial contribution to existing sequence resources; these findings may be useful to accelerate insecticide development as well as insecticide resistance research on this insect.

## Supplementary Materials

Supplementary materials can be found at http://www.mdpi.com/1422-0067/16/09/21873/s1.
