# Supplementary material for: Identification of Genes Putatively Involved in Chitin Metabolism and Insecticide Detoxification in the Rice Leaf Folder (Cnaphalocrocis medinalis) Larvae through Transcriptomic Analysis
Source: Int J Mol Sci. 2015 Sep 10;16(9):21873–96. doi: 10.3390/ijms160921873 (PMC4613286; doi:10.3390/ijms160921873)
Supplement: Supplementary file 1 [file ijms-16-21873-s001.zip › ijms-87573-Supplementary Information/ijms-87573-supplementary figure-for publish.pdf]

Supplementary Information

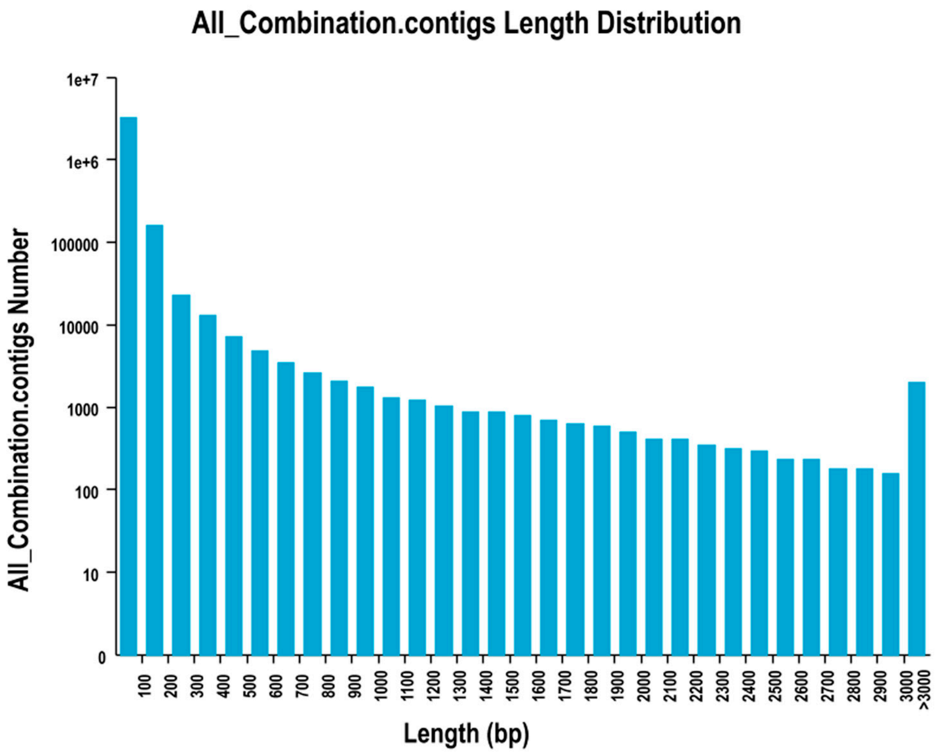

(**A**)

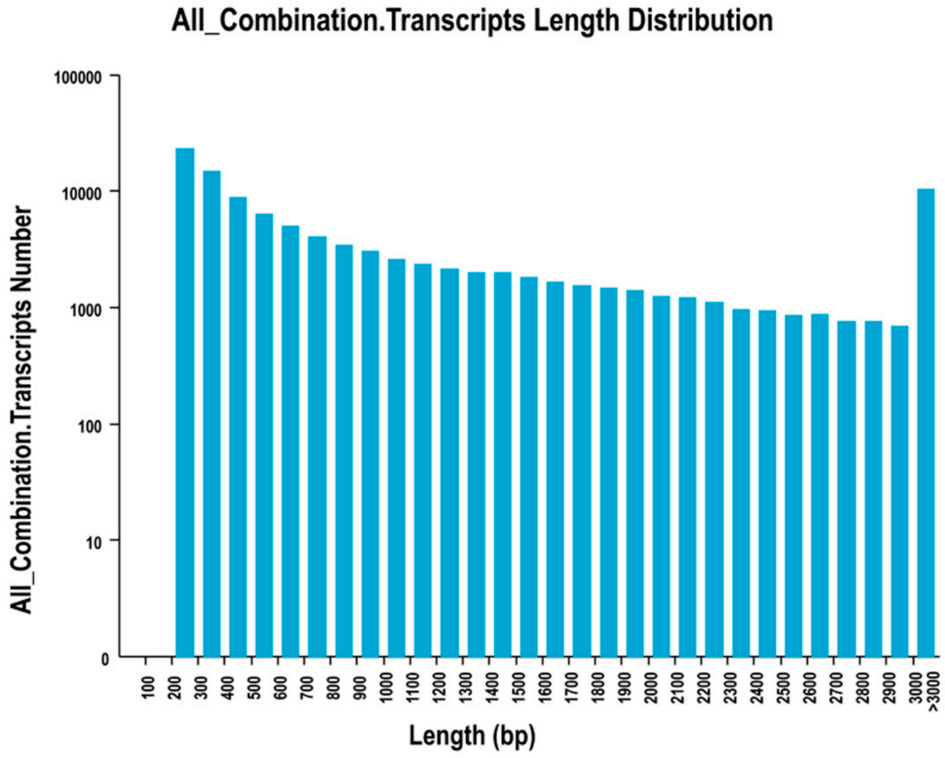

(**B**)

Figure S1. *Cont*.

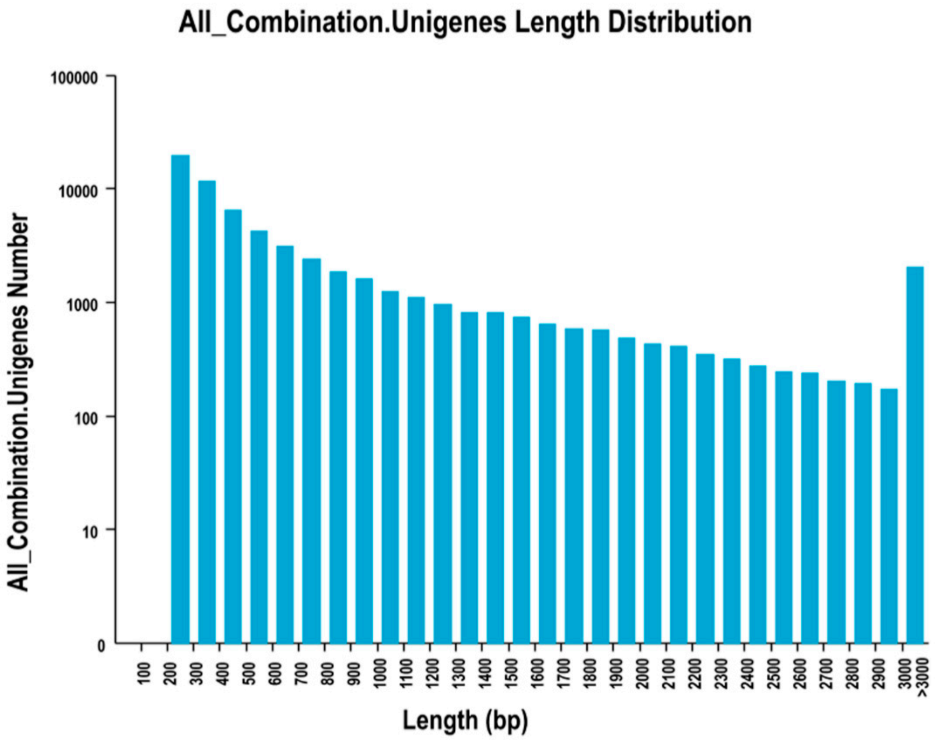

(**C**)

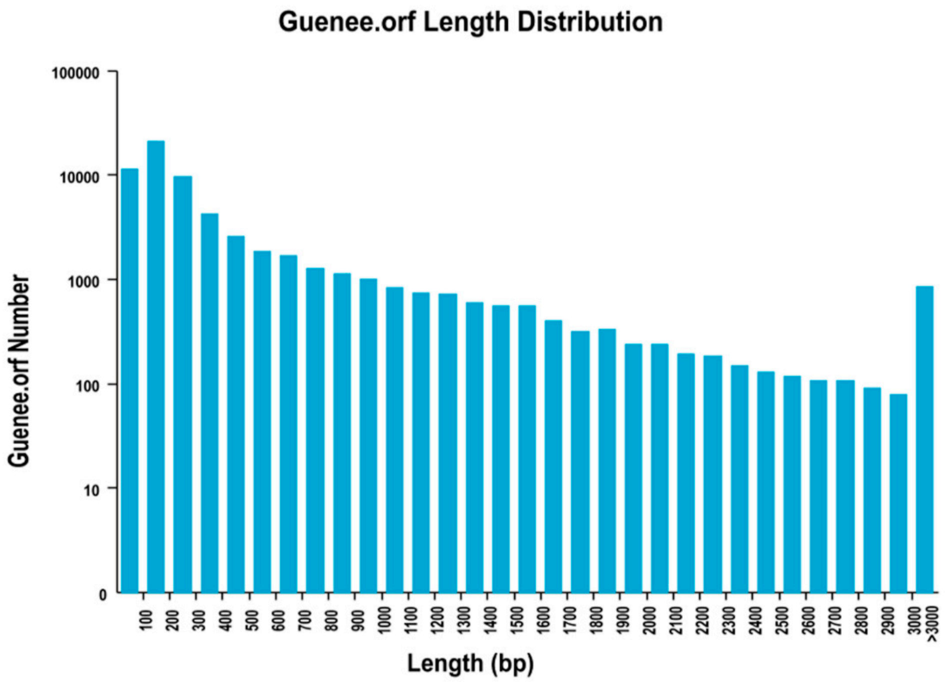

(**D**)

Figure S1. All contigs, transcripts and unigenes distribution of the *C. medinalis* transcriptome sequencing. (**A**) Length distribution of the contigs obtained from do novo assembly of high-quality clean reads; (**B**) Length dietribution of the transcripts produced from further assembly of contigs; (**C**) Size distribution of the unigenes produced from further clustering of transcripts; (**D**) Length distribution of the opening reading frame (ORF). The *x*-axis indicates the length distribution from different range. The *y*-axis indicates the number of unigenes.
